# Linking melanism to brain development: expression of a melanism-related gene in barn owl feather follicles covaries with sleep ontogeny

**DOI:** 10.1186/1742-9994-10-42

**Published:** 2013-07-26

**Authors:** Madeleine F Scriba, Anne-Lyse Ducrest, Isabelle Henry, Alexei L Vyssotski, Niels C Rattenborg, Alexandre Roulin

**Affiliations:** 1 Avian Sleep Group, Max Planck Institute for Ornithology, Eberhard-Gwinner-str.11, Seewiesen 82319, Germany; 2 Department of Ecology and Evolution, University of Lausanne, Lausanne 1015, Switzerland; 3 Institute of Neuroinformatics, University of Zürich and ETH Zürich, Zürich 8057, Switzerland

**Keywords:** REM sleep, Melanocortin, Melanism, Pleiotropy, Ontogeny, Development, Avian

## Abstract

**Background:**

Intra-specific variation in melanocyte pigmentation, common in the animal kingdom, has caught the eye of naturalists and biologists for centuries. In vertebrates, dark, eumelanin pigmentation is often genetically determined and associated with various behavioral and physiological traits, suggesting that the genes involved in melanism have far reaching pleiotropic effects. The mechanisms linking these traits remain poorly understood, and the potential involvement of developmental processes occurring in the brain early in life has not been investigated. We examined the ontogeny of rapid eye movement (REM) sleep, a state involved in brain development, in a wild population of barn owls (*Tyto alba*) exhibiting inter-individual variation in melanism and covarying traits. In addition to sleep, we measured melanistic feather spots and the expression of a gene in the feather follicles implicated in melanism (*PCSK2*).

**Results:**

As in mammals, REM sleep declined with age across a period of brain development in owlets. In addition, inter-individual variation in REM sleep around this developmental trajectory was predicted by variation in *PCSK2* expression in the feather follicles, with individuals expressing higher levels exhibiting a more precocial pattern characterized by less REM sleep. Finally, *PCSK2* expression was positively correlated with feather spotting.

**Conclusions:**

We demonstrate that the pace of brain development, as reflected in age-related changes in REM sleep, covaries with the peripheral activation of the melanocortin system. Given its role in brain development, variation in nestling REM sleep may lead to variation in adult brain organization, and thereby contribute to the behavioral and physiological differences observed between adults expressing different degrees of melanism.

## Introduction

Historically, the potential link between melanism and behavior has been the subject of both scientific and superstitious discussions [1]. In several species, including humans, the degree of melanism is due to genetic variation in the melanocortin 1 receptor (MC1R) that binds the peptide α-melanocyte-stimulating hormone (α-MSH) involved in eumelanin pigment deposition in the skin [2,3]. However, relatively few pleiotropic effects of the *MC1R* gene have been described [2,4]. In contrast, in many other species, a suite of behavioral and physiological traits covary with the degree of melanism [5]. For example, in barn owls, the degree of eumelanin feather spotting covaries with anti-predator responses [6], natal dispersal [7], reproductive activity [8], parasite resistance [9], and the regulation of glucocorticoids [10] and energy [11]. The covariation between melanism and these traits suggests that the genes involved have multiple pleiotropic effects that extend well beyond pigmentation [12].

**Figure 1:**
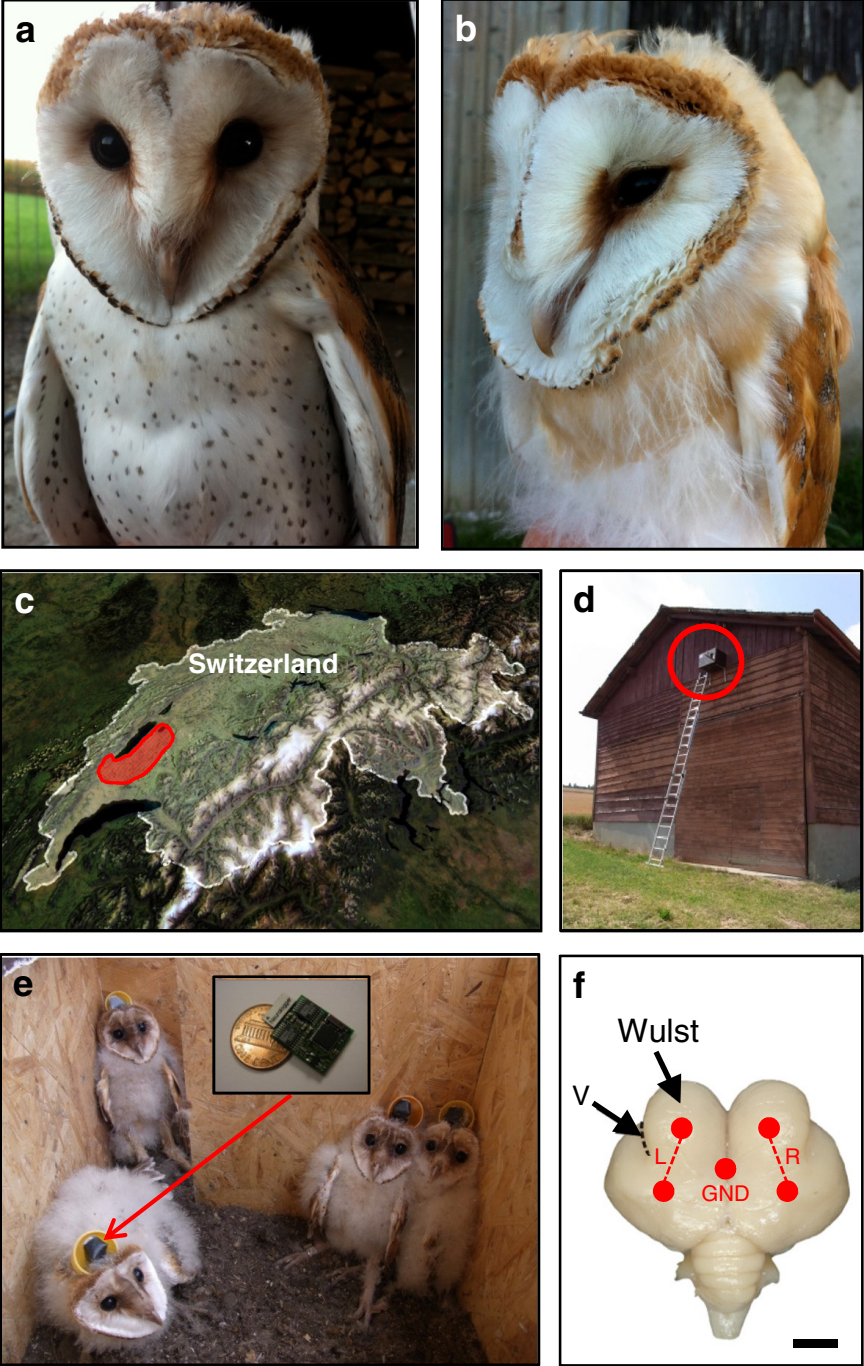
**Study animals, setting, and neurophysiological methods.** Barn owl nestlings displaying high **(a)** and low **(b)** levels of eumelanin spotting, **(c)** satellite image of Switzerland showing the study area shaded in red, **(d)** a nest box hanging on a typical barn, **(e)** nestlings with an EEG/accelerometer logger (inset) attached to their heads, and **(f)** dorsal view of an adult barn owl brain (anterior up) showing the relative location of electrodes (red spots) and bipolar EEG derivations (red dashed lines) for the left (L) and right (R) hemispheres (bar = 0.5 cm); the vallecula groove (V) marks the lateral boundary of the hyperpallium, or *Wulst*. a and b, images by AR; c, image from NASA; d and e, images by MFS (inset by ALV); and f, modified from Martin *et al.*[19] with permission.

The mechanisms linking melanism to these behavioral and physiological traits remain unresolved [5]. Genetic variation in the regulation of hormone and neurotransmitter levels may have direct effects on melanism and other phenotypes in adults [13]. In addition, genetic variation in hormone and neurotransmitter levels might affect adult phenotypes indirectly by acting on developmental processes early in life that influence brain organization. Conversely, genetically determined variation in developmental processes occurring in the brain might determine how hormones and neurotransmitters are regulated in adults. As a first step toward understanding whether developmental processes occurring in the brain contribute to behavioral and physiological differences associated with melanism in adults, we investigated whether the level of a gene involved in melanism expressed in the skin and feathers covaries, not only with peripheral pigmentation, but also with developmental processes occurring in the central nervous system. Instead of following the traditional genetic approach of studying inbred albino and pigmented rodent strains in captivity [14,15], we studied naturally occurring variation in melanism in barn owls in the wild (Figure 1a-d) where they are subject to natural selection, a key to understanding the evolution of such biological traits [16]. To measure ongoing brain development, we focused on sleep because it provides a window into the developing brain [17], which can be viewed using minimally-invasive electroencephalographic (EEG) methods in the wild [18] (Figure 1e,f).

Mammals and birds exhibit two types of sleep, rapid eye movement (REM) and non-REM sleep distinguished from each other and wakefulness using a combination of behavioral and neurophysiological features [20]. In contrast to non-REM sleep, the EEG during REM sleep shows a pattern remarkably similar to that occurring during wakefulness. Despite this awake-like brain activity, the animal is behaviorally asleep with closed eyes and minimal awareness of its surroundings. In mammals, the amount of time spent in REM sleep is highest in young animals, and then gradually declines to adult levels [21-24]. In altricial mammals – those born with relatively immature brains and dependent upon parental care – this occurs before and after birth [21-24], whereas in precocial mammals – those born with relatively mature brains and less dependent upon parental care – the decline in REM sleep occurs primarily before birth [25-29]. Similar age-related declines in REM sleep have been reported in birds [30-32], but the evidence remains contradictory across studies [33] and confounded by factors other than age that might influence REM sleep (e.g., temperature [34], *see* Discussion). In mammals, the age-related decline in REM sleep and subsequent experimental work [17,35] suggest that the awake-like brain activity occurring during REM sleep plays a role in directing brain development [21]. However, the exact role that REM sleep plays in brain development remains unresolved.

The aims of this study were to determine whether the ontogeny of avian sleep follows a mammalian-like trajectory characterized by an age-related decline in REM sleep and to examine whether variation in this developmental process occurring in the brain covaries with components of the melanocortin system expressed in the periphery. For the later we measured in the feather follicles of barn owl nestlings the expression of *PCSK2* – the gene that encodes the proprotein convertase subtilisin/kexin type 2 (PCSK2) responsible for α-MSH synthesis [36] – and its relationship to eumelanin-based feather spotting. Herein we find that 1) REM sleep declines with age in owls, as in mammals, 2) variation around this developmental trajectory is predicted by the expression of *PCSK2* in feather follicles, and 3) *PCSK2* expression predicts eumelanin-based feather spotting. Collectively, these findings demonstrate an unprecedented link between melanism and developmental processes occurring in the brain.

## Results

### Sleep ontogeny

**Figure 2:**
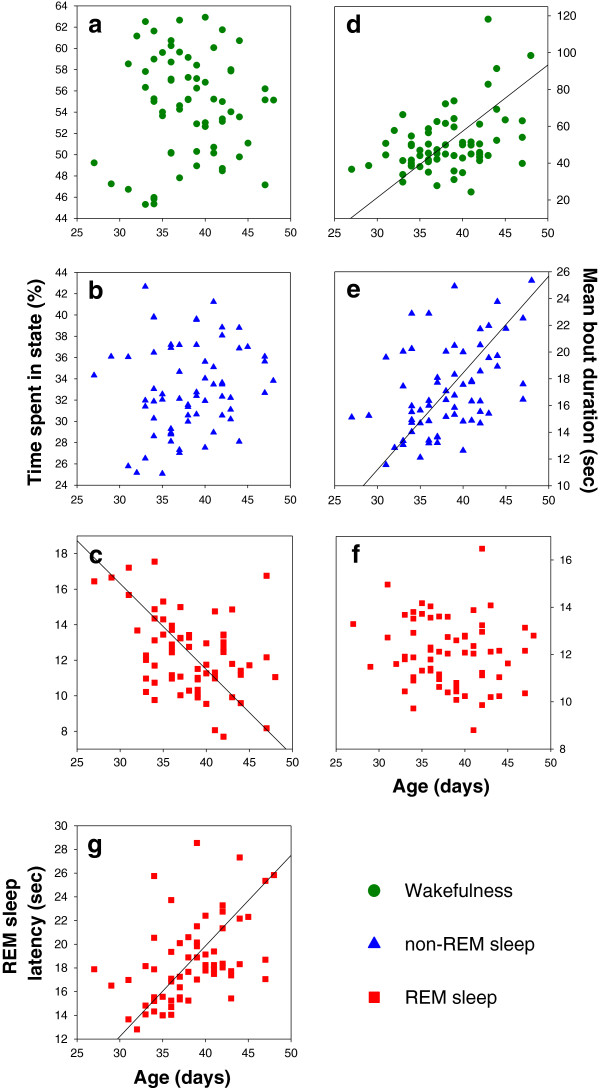
**Time spent in sleep-wakefulness states and mean bout durations are age-dependent.** Time spent in **(a)** wakefulness, **(b)** non-REM sleep, and **(c)** REM sleep, mean bout durations for **(d)** wakefulness, **(e)** non-REM sleep, and **(f)** REM sleep, and **(g)** mean REM sleep latency (*n* = 66). The following correlations with age were statistically significant: duration of bouts of wakefulness (Pearson’s correlation: *r* = 0.41, *p* = 0.0006) and non-REM sleep (*r* = 0.46, *p* = 0.0001), time spent in REM sleep (*r* = -0.40, *p* = 0.0008), and mean REM sleep latency (*r* = 0.47, *p* < 0.0001). None of the other variables correlated significantly with age (*p*-values > 0.18). Similar results were obtained when we calculated mean values for each nest.

The 66 barn owl nestlings were awake approximately half of the time (54.6%, range: 45.3 – 62.9%), in non-REM sleep 33.0% (25.1 – 42.7%), and in REM sleep 12.4% (7.7 – 17.6%). The mean duration of wakefulness bouts was 50.7 ± 2.0 (24.3 – 118.2) sec, of non-REM sleep 17.1 ± 0.4 (11.6 – 25.3) sec, and of REM sleep 12.0 ± 0.2 (8.8 – 16.5) sec. Although the time spent in wakefulness and non-REM sleep did not change significantly with age (Table 1, Figure 2a,b), the duration of bouts of wakefulness and non-REM sleep increased with age (Table 1, Figure 2d,e). Because wakefulness and non-REM sleep mean bout durations were correlated (*r* = 0.63, *n* = 66, *p* < 0.0001), we tested whether the relationship between age and non-REM sleep mean bout duration was confounded by wakefulness mean bout duration in a multiple regression analysis. This was not the case as the relationship between age and non-REM sleep mean bout duration was still significant (*F*_1,63_ = 5.31, *p* = 0.025). In contrast, the relationship between age and wakefulness mean bout duration was no longer significant when non-REM sleep mean bout duration was added in the same model (*F*_1,63_ = 2.11, *p* = 0.15). This suggests that the relationship between wakefulness mean bout duration and age is mediated indirectly by the relationship between age and non-REM sleep mean bout duration*.*

**Table 1:** **Relationships between sleep-wakefulness, age, and *PCSK2 *expression**

	**Wakefulness**		**Non-REM sleep**		**REM sleep**		
	**Percent**	**Bout Duration**	**Percent**	**Bout Duration**	**Percent**	**Bout Duration**	**Latency**
**Age (sleep)**	*F*_1,48.96_ = 0.02	***F***_**1,58.75**_** = 10.69**	*F*_1,43.15_ = 2.48	***F***_**1,52.88**_** = 11.83**	***F***_**1,55.09 **_**= 5.87**	*F*_1,46.09_ = 0.001	***F***_**1,61.52**_** = 19.46**
	*p* = 0.89	***p* = 0.002**	*p* = 0.12	***p* = 0.001**	***p* = 0.019**	*p* = 0.97	***p* < 0.0001**
**Age ( *PCSK2 *)**	*F*_1,54.71_ = 0.05	*F*_1,52.38_ = 0.05	*F*_1,53.97_ = 0.005	*F*_1,52.13_ = 0.003	*F*1,52.28 = 0.39	*F*_1,56.95_ = 0.40	*F*_1,51.86_ = 0.002
	*p* = 0.83	*p* = 0.82	*p* = 0.95	*p* = 0.96	*p* = 0.53	*p* = 0.53	*p* = 0.97
***PCSK2***	*F*_1,57.86_ = 0.47	*F*_1,56.71_ = 1.19	***F***_**1,58.22**_** = 5.14**	*F*_1,57.95_ = 3.66	***F***_**1,57.36**_** = 6.39**	***F***_**1,58.91**_** = 8.49**	*F*_1,57.92_ = 2.67
	*p* = 0.49	*p* = 0.28	***p* = 0.027**	*p* = 0.06	***p* = 0.014**	***p* = 0.005**	*p* = 0.11
**Sex**	*F*_1,52.46_ = 0.0001	*F*_1,52.96_ = 0.03	*F*_1,49.1_ = 0.002	*F*_1,53.64_ = 0.02	*F*_1,53.00_ = 0.10	*F*_1,54.10_ = 0.21	*F*_1,54.83_ = 0.26
	*p* = 0.99	*p* = 0.88	*p* = 0.97	*p* = 0.88	*p* = 0.75	*p* = 0.65	*p* = 0.61
**Date**	*F*_1,30.2_ = 2.49	*F*_1,26.43_ = 1.25	*F*_1,25.28 _= 1.97	*F*_1,23.86_ = 0.03	*F*_1,25.72_ = 1.52	*F*_1,29.38 _= 2.09	*F*_1,22.89_ = 0.06
	*p* = 0.13	*p* = 0.27	*p* = 0.17	*p* = 0.87	*p* = 0.23	*p* = 0.16	*p* = 0.81
**Temperature (mean)**	*F*_1,33.29_ = 0.27	*F*_1,33.49_ = 0.12	*F*_1,30.78_ = 0.14	*F*_1,32.94_ = 0.50	*F*_1,36.84_ = 2.16	*F*_1,30.47_ = 0.003	*F*_1,32.81_ = 0.81
	*p* = 0.60	*p* = 0.73	*p* = 0.71	*p* = 0.49	*p* = 0.15	*p* = 0.96	*p* = 0.37
**Nest (% variance)**	15.7	14.5	1.1	14.4	22.3	8.5	22.3

Linear mixed models for the relationships between the percentage of time spent in and mean bout duration of each state, and expression of *PCSK2* in feather follicles. For REM sleep, the mean latency from sleep onset is also shown. Nest of rearing was set as a random variable. The variables “Age (sleep)” and “Age (*PCSK2*)” are the ages when we measured nestling sleep and *PCSK2* expression, respectively. Significant results of final models are highlighted in bold. The other variables were backward removed starting with the least significant ones. Also shown is the percentage of variance explained by the random factor nest of rearing. This value indicates the extent to which sleep variables of different nest-mates are similar. Mean temperature did not correlate with any sleep variable (*see also* Additional file 1: Figure S3).

Time spent in REM sleep declined with age (Table 1, Figure 2c), a relationship that was even stronger when REM sleep was expressed as a percentage of the total time spent sleeping (Pearson’s correlation: *r* = -0.44, *p* = 0.0002). Because the duration of REM sleep bouts did not change with age (Table 1, Figure 2f), this age-related decline in time spent in REM sleep reflects a reduced propensity to initiate bouts of REM sleep. This propensity was also reflected in a significant age-related increase in the time from sleep onset to the onset of REM sleep (i.e., REM sleep latency) (Table 1, Figure 2g).

### Relationship between sleep ontogeny and *PCSK2* expression

**Figure 3:**
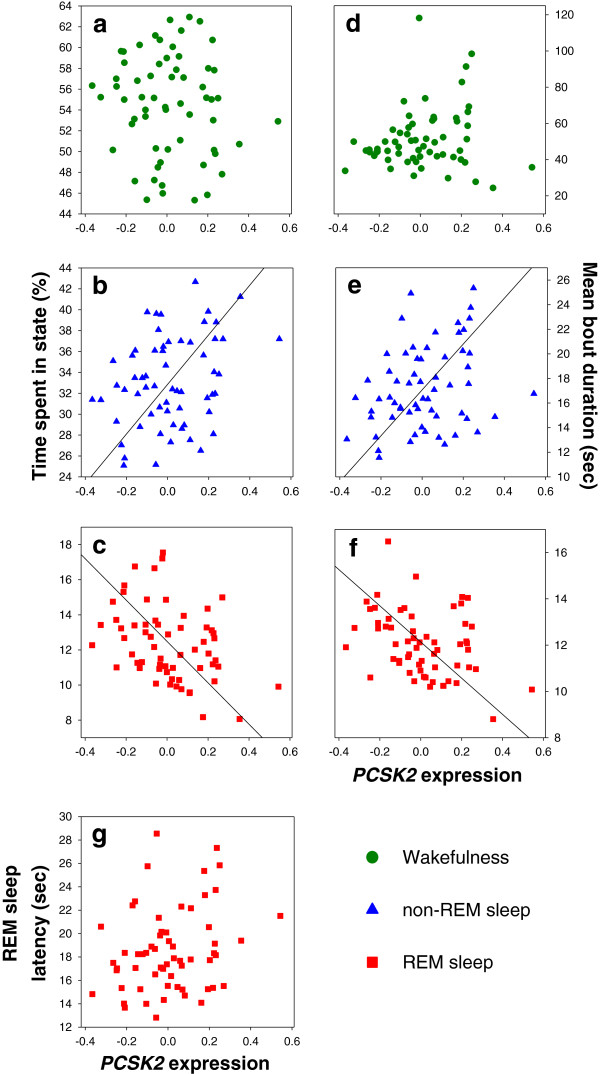
**Time spent in sleep-wakefulness states and mean bout durations correlate with *PCSK2 *expression in feather follicles.** Time spent in **(a)** wakefulness, **(b)** non-REM sleep, and **(c)** REM sleep, mean bout durations for **(d)** wakefulness, **(e)** non-REM sleep, and **(f)** REM sleep and **(g)** mean REM sleep latency (*n* = 61). The following correlations with *PCSK2* expression were statistically significant: time spent in non-REM sleep (Pearson’s correlation: *r* = 0.30, *p* = 0.019) and duration of bouts of non-REM sleep (*r* = 0.29, *p* = 0.023; *see also* analysis in Table 1 where this relationship is only a trend), time spent in REM sleep (*r* = -0.36, *p* = 0.005), and duration of bouts of REM sleep (*r* = -0.35, *p* = 0.006). None of the other variables correlated significantly with *PCSK2* expression (*p*-values > 0.23). Similar results were obtained when we calculated mean values for each nest.

We next examined the relationship between these ontogenetic patterns occurring in the brain and *PCSK2* expression in feather follicles. The level of *PCSK2* expression increased with nestling age (linear mixed model with the random variable nest of rearing explaining 22.36% of the variance: *F*_1,122.9_ = 6.50, *p* = 0.012), but was not associated with nestling sex (*F*_1,121.5_ = 0.34, *p* = 0.56), date (*F*_1,38.35_ = 0.02, *p* = 0.89), and time of the day when feathers were collected (*F*_1,72.77_ = 0.71, *p* = 0.40; all two-way interactions with sex were not significant, *p* > 0.15). Although the time spent in wakefulness and the duration of wakefulness bouts were not correlated with *PCSK2* expression (Table 1, Figure 3a,d), time spent in non-REM sleep and the duration of non-REM sleep bouts were positively correlated with *PCSK2* expression (Table 1, Figure 3b,e). In contrast, the time spent in REM sleep and the duration of REM sleep bouts were strongly negatively correlated with *PCSK2* expression (Table 1, Figure 3c,f), suggesting that part of the age-related decline in REM sleep was associated with *PCSK2* expression. Finally, REM sleep latency was not correlated with *PCSK2* expression (Table 1, Figure 3g). Importantly, none of the sleep-wakefulness variables was correlated with the age when *PCSK2* was measured (Table 1), which differed from the age when sleep was measured, indicating that the associations between these variables and *PCSK2* were not due to simple covariation between age and *PCSK2* expression. Collectively, these findings suggest that the propensity to initiate bouts of REM sleep declines with age, whereas the propensity to terminate bouts of REM sleep increases with *PCSK2* expression. Thus, age and *PCSK2* expression, or covarying processes, influence different aspects of REM sleep during development.

### Genetic basis of eumelanin feather spottiness

On average nestlings displayed 40.5 ± 1.6 spots (range: 0 – 76) measuring 1.45 ± 0.05 mm (range: 0 – 2.38), breeding females 44.5 ± 2.5 spots (range: 14 – 76) measuring 1.74 ± 0.09 mm (range: 0.86 – 3.00) and breeding males 38.4 ± 4.4 spots (range: 0 – 88) measuring 1.27 ± 0.10 mm (range: 0 – 2.38). There was no evidence of assortative mating with respect to spotting (Pearson’s correlations between mother and father spottiness: *r* = 0.02, *p* = 0.94). Furthermore, biological and foster parents did not resemble each other with respect to plumage traits (Pearson’s correlations: *p*-values > 0.31). In line with the assumption that the expression of feather spots is under genetic control, spottiness in the cross-fostered nestlings was strongly correlated with maternal (Pearson’s correlation based on mean sibling values: *r* = 0.49, *n* = 24, *p* = 0.015) and paternal spottiness (*r* = 0.53, *n* = 18, *p* = 0.023).

### Feather *PCSK2* expression and eumelanin spottiness

**Figure 4:**
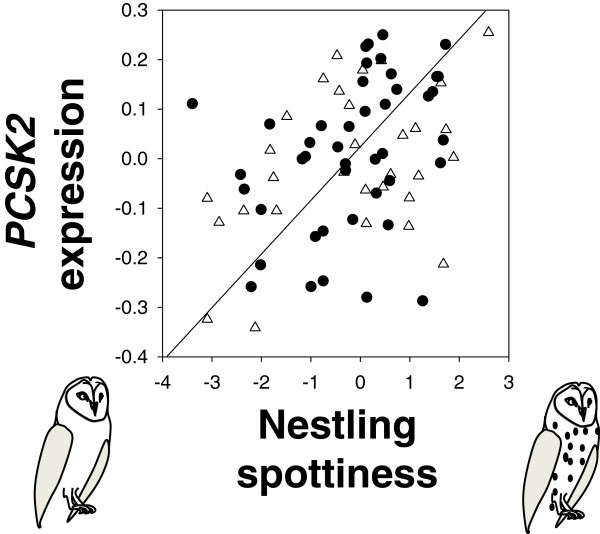
**Nestling spottiness correlates positively with *PCSK2 *expression in feather follicles.** Data for male (triangles) and female (circles) cross-fostered nestlings. *PCSK2* expression was positively correlated with nestling spottiness (Pearson’s correlation: *r* = 0.39, *n* = 75, *p* = 0.0006).

In cross-fostered nestlings, *PCSK2* expression was positively associated with nestling eumelanin spottiness (Figure 4; linear mixed model with nest of rearing as the random variable: nestling spottiness: *F*_1,49.58_ = 8.71, *p* = 0.005; age when *PCSK2* was measured: *F*_1,70.8_ = 1.73, *p* = 0.20; interaction: *F*_1,66.69_ = 0.39, *p* = 0.53). Thus, *PCSK2* expression in feather follicles is correlated with both the ontogeny of sleep and melanic feather spotting in nestlings.

## Discussion

**Figure 5:**
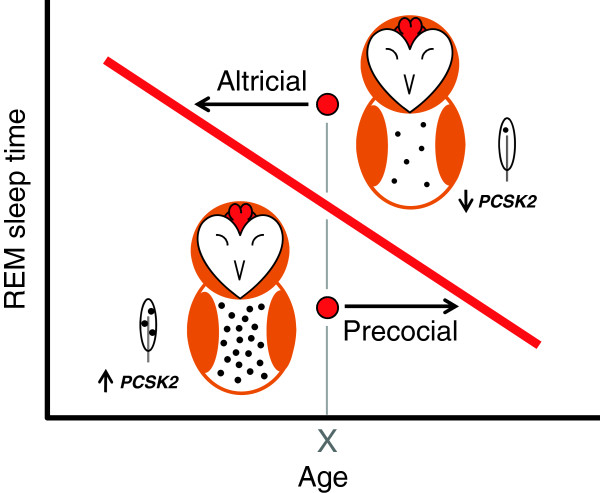
**Summary illustration.** Interrelationship between the age-related decline in REM sleep (red line), and *PCSK2* expression in the feather follicles and associated melanic feather spotting (•). For a given age (X), owlets with low *PCSK2* expression (top) had low amounts of spotting and high amounts of REM sleep, whereas owlets with high *PCSK2* expression (bottom) had high amounts of spotting and low amounts of REM sleep. In this respect, owlets expressing lower amounts of *PCSK2* exhibited REM sleep amounts typical of younger birds, an altricial pattern of brain development, whereas owlets expressing higher amounts of *PCSK2* exhibited REM sleep amounts typical of older birds, a precocial pattern of brain development.

In this study, we demonstrate a previously unrecognized link between developmental processes occurring in the central nervous system and processes involved in melanism occurring in the periphery. Sleep ontogeny in owlets undergoing a period of brain growth followed a mammalian-like pattern [24] characterized by an age-related increase in the duration of bouts of wakefulness and non-REM sleep. Also as in mammals, REM sleep time and latency decreased and increased, respectively, as the owlets aged [24]. For REM sleep, in particular, this developmental trajectory covaried strongly with *PCSK2* expression in the feather follicles, with owlets expressing higher *PCSK2* showing lower amounts of REM sleep, a more precocial pattern (Figure 5). Finally, eumelanin-based spottiness was genetically determined and positively correlated with *PCSK2* expression in feather follicles.

### Sleep ontogeny: comparison with prior research

While it is often stated that the amount of REM sleep is higher in young birds when compared to adults [37], a review of the literature reveals that this issue is far from resolved. Prior to our study, the ontogeny of REM sleep had been examined in only two avian species, the precocial chicken (*Gallus gallus domesticus*) and the altricial magpie (*Pica pica*). However, the results remain contradictory and/or confounded by environmental factors known to influence the expression of REM sleep. While REM sleep can be first identified 1 – 2 d before chicken embryos hatch [38,39], age-related changes in REM sleep have been examined primarily following hatching. Several studies have reported that REM sleep as a % of total sleep time (%REM sleep) declines with age from higher amounts occurring shortly (0 – 8 h) after hatching [30,31,40,41]. However, others have reported *lower* %REM sleep in newly hatched chicks [33]. Multiple potentially interacting factors may contribute to the variability in reports of REM sleep ontogeny in chickens. As noted by Schlehuber *et al.*[33], when compared to older chicks, EEG activity during REM sleep was less differentiated from that occurring during non-REM sleep in newly hatched chicks (*see also*[42]). As a result, this may have led to different interpretations as to what constitutes REM sleep. A similar issue has also contributed to the debate over when REM and non-REM sleep are first distinguishable from one another in mammals [43,44]. In addition, in chickens, the amount of REM sleep recorded at different time points in the various studies could have been influenced by, 1) the time from surgery to the first recordings (i.e., short in newly hatched chicks), 2) the brain regions recorded, 3) the social conditions (isolated or in groups) in the recording environment, and 4) exposure to potential imprinting stimuli, such as the experimenter. Indeed, after most ontogenetic work on chicks had been conducted, it was shown that exposure to a visual imprinting stimulus after hatching caused an increase in REM sleep [45]. Finally, a general concern regarding examining the ontogeny of REM sleep in recently hatched chicks is the possibility that the process of hatching itself causes a temporary increase or decrease in REM sleep that masks its true relationship (or lack thereof) with brain development. This is potentially particularly problematic in precocial species, such as chickens, if they exhibit a mammalian-like pattern of attaining adult REM sleep levels shortly after hatching.

In the only prior study examining the development of sleep in an altricial bird, Szymczak reported lower %REM sleep in adult (1 – 2-year-old) magpies when compared to independent juveniles (3.5 – 4.0-month-old) [32], a pattern seemingly comparable to that observed in altricial mammals. However, the juveniles were recorded under warmer ambient temperatures than the adults. This difference confounds the interpretation of these results because, as in mammals, avian REM sleep is suppressed by low ambient temperatures [46,47]. Indeed, in a subsequent study, Szymczak showed that the low temperatures experienced by the adult magpies in the earlier study also suppress REM sleep in this species [34]. Given these concerns regarding prior ontogenetic studies in chickens and magpies, our finding of a temperature-independent (Table 1), age-related decline in REM sleep in barn owl nestlings is to our knowledge the first unequivocal evidence for this mammalian-like pattern in birds. The presence of this pattern in mammals and birds suggests that it reflects a fundamental aspect of sleep in animals that exhibit REM sleep.

### Linking sleep ontogeny to peripheral *PCSK2* expression

How might *PCSK2* expression in feather follicles be related to the ontogeny of sleep in the brain? Although peptides produced by PCSK2 processing in the follicle (α-MSH and corticotropin-like intermediate lobe peptide (CLIP)) might reach the brain [48] and thereby influence regions regulating sleep, the effects that these compounds have on sleep when injected intracerebralventricularly (icv) in adult mammals are inconsistent with this bottom-up scenario. Whereas higher *PCSK2* expression was associated with less REM and more non-REM sleep in owls, both sleep states decreased and wakefulness increased following α-MSH (0.5 – 50 μg) injection in rabbits [49]. In rats, although a low dose (1 ng) of α-MSH increased non-REM sleep [50], higher doses (0.05 – 6.0 μg) had no affect on sleep or wakefulness [51]. Moreover, in all studies, REM sleep increased following injection of CLIP in rats [50,52-54]. In addition, whereas the duration of bouts of REM sleep decreased with increasing *PCSK2* expression in owls, CLIP increased the duration of REM sleep bouts in rats [53]. To the extent that these findings in adult mammals receiving icv injections of α-MSH and CLIP translate to young owls, they do not support the notion that the production of these peptides in the follicles accounts for the relationships observed between *PCSK2* expression and sleep.

Alternatively, increased levels of monoamine neurotransmitters known to suppress REM sleep in mammals and birds [55] might also increase the release of hormones from the hypothalamic-pituitary-adrenal (HPA) and -thyroid (HPT) axes, resulting in activation of peripheral components of the melanocortin system [13]. Because the HPA hormone adrenocorticotropic hormone (ACTH) is the precursor that PCSK2 processes into α-MSH, ACTH released into the circulation may reach the follicle and lead to increased *PCSK2* expression. In addition to the potential direct effects that monoamine neurotransmitter levels might have on brain regions controlling REM sleep, they may also influence REM sleep amounts indirectly via activation of the HPT axis. Thyroid hormones produced in the thyroid gland (via the HPT axis) play an important role in brain development and its timing [56-60]. Consequently, increased levels of thyroid hormones might accelerate brain development, and thereby account for the precocial age-related decline in REM sleep observed in owlets with high levels of peripheral *PCSK2* expression. Finally, given that PCSK2 is involved in processing neuropeptides in the periphery (e.g., insulin) and brain (e.g., thyroid releasing hormone) involved in brain development [61-63], the relationship between peripheral *PCSK2* expression and REM sleep might arise if genetic polymorphisms in transcription factor binding sequences of *PCSK2*, or transcription factors regulating the expression [64], stability, or splicing of *PCSK2* affect its expression throughout the body and brain in a coordinated manner. The finding that the relationship between *PCSK2* expression and feather spotting did not vary with age (the interaction between nestling spottiness and nestling age on *PCSK2* expression was not significant, *p* = 0.86) suggests that *PCSK2* expression was not locally regulated exclusively with regard to the feather formation process, but instead may have been determined by global regulatory mechanisms both during and outside the period of feather formation. Regardless of the exact mechanism(s) responsible for the strong link between peripheral *PCSK2* expression and REM sleep, this variation in the amount of REM sleep likely reflects and/or mediates developmental processes occurring in the brain.

### Evolutionary considerations

The covariation between pigmentation, peripheral *PCSK2* expression, and sleep ontogeny observed in barn owls in nature may be maintained by trade-offs related to varying the pace of brain development. Owlets that expressed higher levels of *PCSK2* in the feather follicles exhibited REM sleep amounts typical of older owlets. To the extent that the amount of REM sleep predicts brain development in owls, this suggests that brain development is more precocial in these individuals. Among birds, precocial species tend to have smaller brains for their size than altricial species [65], and species with bigger brains engage in more innovative feeding behaviors [66] and survive longer in nature [67]. If such relationships exist in barn owls, the apparent cost of precocial brain development may be balanced by other beneficial traits associated with melanism, including enhanced anti-inflammatory responses and better regulation of the balance between food intake and energy expenditure [12]. Ultimately, brain imaging studies are needed to establish whether and how this link between the periphery and the developing brain influences adult brain organization, behavior, and physiology. Finally, in addition to potentially influencing adult behavior and physiology via brain development, variation in REM sleep may also influence these traits more directly if it persists into adulthood [68].

## Conclusions

We demonstrate that the pace of brain development, as reflected in age-related changes in sleep, covaries with the peripheral activation of the melanocortin system. Although the mechanisms linking these processes remain unresolved, our findings suggest a previously unrecognized pathway that may account for the link between melanism and a variety of behavioral and physiological phenotypes in adults. Specifically, variation in developmental processes occurring in the brain linked to melanism may influence adult brain organization and thereby its regulation of behavior and physiology. In this respect, our findings move the field forward by establishing a new framework for investigating how and why pigmentation is often predictive of other seemingly unrelated phenotypes. More generally, our findings expand our understanding of the breadth of the interface between the central nervous system and the rest of the body.

## Methods and materials

### General procedures

In 2011 we studied a population of free-living barn owls in Switzerland (Figure 1c). Our aim was to examine potential mechanisms that link a peripheral trait (i.e., eumelanin-based black feather spots; Figure 1a,b) with developmental processes taking place in the central nervous system (i.e., sleep), not only at the precise time when coloration is produced, but also outside this period, as many of the associations between spottiness and other traits occur outside the period of feather formation [12]. For this reason, we measured the expression level of *PCSK2* in the feather follicles [36] and feather spottiness in 75 nestlings (31 males, 44 females) at an age (46.6 ± 0.5 d; range: 37 – 59) when feather spots had already been produced (20 – 35 d); sex was determined using molecular markers [69], and the day of hatching was determined by measuring the length of the left flattened wing from the bird’s wrist to the tip of the longest primary soon after hatching [70]. For ethical reasons, we measured sleep at an earlier age to allow time for the small patch of feathers removed from the head for the EEG recordings to re-grow before fledging. Our approach did not appear to have any long-term adverse effects on the owls, as all of the birds fledged and recruitment into the breeding population in the following year (2012) was actually higher in owls that had their sleep recorded (26.3%), than in those that had not (19.0%). This research was approved by the Service Vétérinaire du Canton de Vaud (license 1508.5), and adhered to the National Institutes of Health standards regarding the care and use of animals in research. Statistical analyses were performed with the software program JMP 8.0.1. Tests were two-tailed with *p*-values < 0.05 considered significant. Means are quoted ± se.

### Rearing environment

The birds were recorded in broods of 2 – 8 individuals in their nest boxes (Figure 1d,e). Each nest box (62 × 56 × 37 cm) was hung (about 8 meters up) on the external wall of a barn surrounded by farmland and various habitats [71]. A 10 × 15 cm door allowed entry of some natural light during the day. Presumably, the nestlings could also hear natural and human made noises. Ambient daily temperatures were obtained from the Swiss Meteorological Institute (MeteoSwiss) at a station in Payerne which is situated in the center of the study area. The mean daily temperature was 16.5 ± 0.4°C (range 10.5 – 21.5°C).

### Sleep recordings

We recorded sleep in nestlings (31 males and 35 females from 29 nests) between 27 – 48 d of age (38.2 ± 0.55 d). Nestlings in this age range have open eyes, but are still undergoing a large amount of brain development [72] and remain dependent upon parental provisioning of food. Each nestling was instrumented with a small data logger [73] (Neurologger2, http://www.vyssotski.ch/neurologger2) that recorded EEG activity (band-pass filtered 1 – 70 Hz, first order) and head movements using a minimally-invasive electrode attachment method (*see* Additional file 1) initially developed for use in humans [74], and validated for use in owls [18]. In a subset of birds, simultaneous video recordings were obtained to relate EEG and accelerometer signals to specific behaviors (Additional file 1: Figure S1; Additional file 2: Video S1). EEG recordings were obtained between May and October 2011.

### Behavioral state scoring

The last complete 24-h d of each recording was visually scored in 4 sec epochs for wakefulness, non-REM sleep, and REM sleep by an experienced investigator (NCR) blind to the age and pigmentation of the owls, as well as their *PCSK2* expression levels. The 24-h period usually started several days after the owls were returned to their nest following logger attachment (mean, 65 h 55 min; range, 21 h 15 min – 92 h 29 min). To determine whether variation in this latency influenced sleep, we performed linear mixed models with nest of rearing as the random variable and box-cox transformed latency as the independent variable. Latency was not significantly associated with the time spent in wakefulness, non-REM sleep, or REM sleep, nor the mean bout duration of any state (Pearson’s correlations, *p*-values > 0.11). Consequently, sleep normalized rapidly after return to the nest.

The percentage of the 24-h period spent in each state was calculated for each nestling. In addition, the duration of individual bouts of each state was calculated as the number of consecutive epochs spent in a given state. The mean bout duration was calculated for each state for each bird. Finally, we calculated the REM sleep latency as the elapsed time from sleep onset (≥ 4 sec of non-REM or REM sleep) to the onset of REM sleep (≥ 4 sec) following bouts of wakefulness ≥ 4 sec; i.e., when an owlet went directly from wakefulness to REM sleep the latency was zero. We also examined the REM sleep latency following only longer bouts of wakefulness (28 and 60 sec) and obtained similar results. The mean REM sleep latency was calculated for each nestling. The data presented in Figure 2 is available in Additional file 3.

### Measurement and genetics of eumelanin feather spotting

In nestlings and their genetic parents (*see below*), we measured the number and diameter of eumelanin-based spots within a 60 × 40 mm area of the breast. In nestlings, *PCSK2* expression was measured in the same area. The number of black spots was strongly correlated with mean spot diameter within nestlings, and within breeding females and males (0.88 > *r* > 0.47, *p* < 0.006). For this reason, and because preliminary analyses showed that spot number and diameter were similarly associated with *PCSK2* expression and sleep, we took the first principal component of a principal components analysis including spot number and mean spot diameter. Because *PCSK2* expression did not differ between males and females (*see* Results) and also because females are on average spottier than males [70], we centered these groups separately by z-scoring. The first principal component explained 93.4% of the variance in nestlings (eigenvalue: 1.87), 73.3% in breeding females (eigenvalue: 1.47), and 92.9% in breeding males (eigenvalue: 1.86). We define this first principal component as plumage “spottiness”. The nestlings’ age when the feathers were collected was not associated with spottiness measured in nestlings, nor their biological parents (two separate linear mixed models with nest of rearing as the random variable: *p*-values > 0.14).

The following steps were taken to isolate the genetic contribution to nestling spottiness. Although extra-pair paternity is very low in our population [75], in the present study we verified that the social parents of each offspring were the biological parents using six genetic markers (*Ta204*, *Ta206*, *Ta216*, *Ta310*, *Ta413*, and *Ta414*) [76]. In addition, to control for the potential impact that rearing conditions might have on the measured phenotypes, we performed a cross-fostering experiment at hatching by swapping half of the hatchlings between pairs of nests, thereby allocating genotypes randomly among rearing environments.

### Feather eumelanin spotting and *PCSK2* expression

We measured *PCSK2* expression using standard methods (*see* Additional file 1) in 75 cross-fostered nestlings from 26 families. *PCSK2* expression was measured in 61 of the 66 individuals for which we assessed sleep.

## Abbreviations

ACTH: Adrenocorticotropic hormone; α-MSH: α-melanocyte-stimulating hormone; CLIP: Corticotropin-like intermediate lobe peptide; EEG: Electroencephalogram; HPA: Hypothalamic-pituitary-adrenal; HPT: Hypothalamic-pituitary-thyroid; icv: intracerebralventricularly; MCR1: Melanocortin 1 receptor; MC1R: Gene encoding *MCR1*; non-REM: non-rapid eye movement; PCSK2: Proprotein convertase subtilisin/kexin type 2; PCSK2: Gene encoding PCSK2; REM: Rapid eye movement.

## Competing interests

The authors declare that they have no competing interests.

## Authors’ contributions

AR, A-LD, MFS, and NCR designed the study. MFS collected and NCR scored the neurophysiological data. A-LD quantified gene expression. AR performed the statistical analyses. IH collected biometric data from the owls. ALV developed and provided Neurologger2s and contributed to the figures. AR, A-LD, MFS, and NCR wrote the paper. A-LD and MFS contributed equally, as did AR and NCR. All authors discussed the results and commented on the manuscript. All authors read and approved the final manuscript.

## Supplementary Material

Additional file 1: Table S1 Quantitative PCR primers and probes used to measure *PCSK2* expression in feather follicles. **Figure S1.** Representative EEG and accelerometry recordings showing wakefulness, non-REM sleep, and REM sleep. Eight consecutive minutes are shown with 1 minute per panel (a-h). Additional file 2: Video S1 shows the corresponding behavior. The first minute starts with the owlet preening (a), as reflected in the high-frequency oscillations in the accelerometer recordings and corresponding EEG artifacts. The bird then spends an extended period (>3 min) looking around the box (a-e). The small abrupt changes in the accelerometer recordings correspond to head movements. Low-amplitude, high-frequency EEG activity indicative of wakefulness is evident between the head movements. Upon falling asleep (e), the rapid head movements stop, the eyes close, and the EEG shows high-amplitude, low-frequency waves indicative of non-REM sleep. The remaining panels (f-h) show alternations between non-REM and REM sleep. During REM sleep the EEG shows wakefulness-like activity, but the head remains still or falls gradually, and the bird’s eyes remain closed. Similar behavior occurs in owlets without the EEG logger on their head (see bird on the right in Additional file 2: Video S1 at 16:27:52). **Figure S2.** EEG power density for wakefulness, non-REM sleep, and REM sleep. The three black lines mark significant differences (*p* < 0.05; two-tailed *t*-tests) between wakefulness and non-REM sleep (W vs N), wakefulness and REM sleep (W vs R), and non-REM and REM sleep (N vs R). **Figure S3.** Mean time spent in each state for all owls recorded on a given julian day and the corresponding mean temperature. Click here for file

Additional file 2 **Video S1.** Eight minute video corresponding to the EEG and accelerometry recordings shown in Additional file 1: Figure S1. The bird is positioned in the left half of the frame. A second bird can be seen in the right half of the frame. Click here for file

Additional file 3 **File containing sleep data from Figure** 2. Click here for file
