# Supplementary material for: Linking melanism to brain development: expression of a melanism-related gene in barn owl feather follicles covaries with sleep ontogeny
Source: Front Zool. 2013 Jul 26;10:42. doi: 10.1186/1742-9994-10-42 (PMC3734112; doi:10.1186/1742-9994-10-42)
Supplement: Additional file 1: Table S1 — Quantitative PCR primers and probes used to measure PCSK2 expression in feather follicles. Figure S1. Representative EEG and accelerometry recordings showing wakefulness, non-REM sleep, and REM sleep. Eight consecutive minutes are shown with 1 minute per panel (a-h). Additional file 2: Video S1 shows the corresponding behavior. The first minute starts with the owlet preening (a), as reflected in the high-frequency oscillations in the accelerometer recordings and corresponding EEG artifacts. The bird then spends an extended period (>3 min) looking around the box (a-e). The small abrupt changes in the accelerometer recordings correspond to head movements. Low-amplitude, high-frequency EEG activity indicative of wakefulness is evident between the head movements. Upon falling asleep (e), the rapid head movements stop, the eyes close, and the EEG shows high-amplitude, low-frequency waves indicative of non-REM sleep. The remaining panels (f-h) show alternations between non-REM and REM sleep. During REM sleep the EEG shows wakefulness-like activity, but the head remains still or falls gradually, and the bird’s eyes remain closed. Similar behavior occurs in owlets without the EEG logger on their head (see bird on the right in Additional file 2: Video S1 at 16:27:52). Figure S2. EEG power density for wakefulness, non-REM sleep, and REM sleep. The three black lines mark significant differences (p < 0.05; two-tailed t-tests) between wakefulness and non-REM sleep (W vs N), wakefulness and REM sleep (W vs R), and non-REM and REM sleep (N vs R). Figure S3. Mean time spent in each state for all owls recorded on a given julian day and the corresponding mean temperature. [file 1742-9994-10-42-S1.docx]

**Supplementary Material**

Scriba *et al*. Linking melanism to brain development: Expression of a melanism-related gene in barn owl feather follicles predicts sleep ontogeny*. Frontiers in Zoology*

**Supplementary Methods and Materials**

**EEG recordings**

A few feathers on top of the head were cut, the skin was anesthetized (Gingicain, Tetracain 754 mg / 65 g), and 5 wire electrodes (stainless steel medical wire, diameter: 0.13 mm, 2 mm of the insulation exposed; Cooner Wire, Owensmouth, Chatsworth, CA) were inserted with a 23-gauge hypodermic needle under the superficial layers of skin. The electrodes were placed over each brain hemisphere, positioned over the posterior part of the visual hyperpallium (*Wulst*), a primary visual area in some respects homologous to the mammalian primary visual cortex [77], and referenced to a posterior electrode placed over the caudal nidopallium (Figure 1f). A 5th electrode was centered between the other electrodes and served as a ground. The electrodes were held in place with superglue and connected to the data logger. Additionally, the data logger incorporated an accelerometer (LIS302DLH, STMicroelectronics) which recorded the acceleration of the head in all 3 dimensions. The total weight of the device (5 g) was at most 2.1 % of the bird’s body weight. The data logger was glued to the head and protected by a piece of plastic (Figure 1e). Bipolar EEG signals from the left and right hemispheres (Figure 1f) and the 3 acceleration signals were sampled and recorded at 200 Hz for up to 5 d for each nestling.

During wakefulness, movement artifacts obscured the EEG during very active behaviours (e.g., preening, feeding, and regurgitating hair and bone pellets), as shown in the video and accelerometer recordings (Figure S1a; Video S1). However, when the birds only exhibited small, rapid (jerky) head movements or were briefly still (approximately 10 % of the time), the EEG showed low-amplitude, high-frequency activity typical of wakefulness; these still periods were used for the EEG spectral analysis of this state (*see below*). As in other birds [78], the owlets spent very little time awake and still, and instead transitioned to non-REM sleep within seconds of becoming still (Figure S1e). During non-REM and REM sleep the EEG was artifact free > 90 % of the time. During non-REM sleep, the EEG was dominated by high-amplitude, low-frequency (≈ 1.5 – 5 Hz) activity (Figure S1a-h; S2). The head was held motionless, in an upright position, or, in some cases, when preceded by REM sleep, in the last position attained during that state. As in adult barn owls [18], the nestlings’ eyes were usually closed and interhemispheric asymmetries in EEG activity were rarely observed. During REM sleep, the EEG rapidly switched to a pattern similar to that observed during wakefulness; although there were some small yet significant differences between REM sleep and wakefulness (Figure S2). In contrast to wakefulness, a reduction in muscle tone [79, 80] caused the head to fall forward, sideways, or backwards during REM sleep (Video S1). REM sleep-related head movements were occasionally momentarily interrupted by phasic twitching, as in other birds and mammals. Finally, on hotter days the owls panted during non-REM sleep, producing distinct rhythmic accelerometer signals. Panting stopped during each episode of REM sleep, suggesting that, as in other birds and mammals [54, 55], thermoregulatory responses are suspended during REM sleep in owl nestlings in this age range.

**EEG spectral analysis**

The EEG power spectrum was calculated (FFT, Somnologica Science v. 3.3.1) for wakefulness, non-REM sleep, and REM sleep (0.8 – 50 Hz, in 0.4 Hz bins) following established procedures [81, 82]. The left EEG was analyzed for most birds (*n* = 48). In cases where the EEG signal was markedly better in the right hemisphere (*n* = 18), this EEG was analyzed instead. Epochs with artifacts were removed from the analysis. Large movements sometimes caused a change in the overall amplitude of the EEG signal that then remained stable for an extended period. Following such shifts, the relative differences between the three states were always retained. Presumably, this reflects changes in the position of the subcutaneous electrodes relative to the brain resulting from movement of the logger on the birds’ head. While this did not hinder our ability to distinguish between states, it precludes assessments of potential changes in EEG activity over longer time scales related to homeostatic or circadian processes. Because all three states were represented during each signal shift, all otherwise artifact-free epochs were included in the analysis of sleep-related EEG power. For each bird, the mean value was calculated for each frequency bin and state and expressed as a percentage of the 24-h non-REM sleep average across all frequency bins.

***PCSK2* expression**

For the analysis of *PCSK2*, the follicles of breast feathers were collected and immediately frozen in dry ice and stored at -80°C. Feather follicles were grinded in liquid nitrogen and the RNA was extracted with RNAeasy mini kit and DNase I treatment (Qiagen, Hombrechtikon, Switzerland). One aliquot of total RNA was used to assess the quantity of the total RNA with Qubit fluorometer (Life Technologies, Zug, Switzerland) and the quality using the Bioanalyser (Agilent Technologies, Basel, Switzerland). A second DNase treatment was applied to 1 ug of total RNA with 5U DNase I recombinant, RNase-free from bovine pancreas (Roche Diagnostics, Rotkreuz, Switzerland), 10U RNasin Ribonuclease Inhibitor (Promega AG, Duebendorf, Switzerland), 10 mM Tris-HCl pH 8.0, 0.5 mM MgCl_2_, 1 mM DTT in 10 ul for 30 min at 37°C, followed by inactivation at 65°C for 10 min. We reverse transcribed 100 ng of DNase I treated RNA in 20 ul with 50 pmol of random hexamer primers, and 200U of Superscript III according to the manufacturer’s protocol (Life Technologies, Zug, Switzerland). We then precipitated the cDNA with one volume of 5M NH_4_OAc (pH 8.0) and 2.5 volumes of cold ethanol 95%, the pellet was resuspended in one volume of 10 mM Tris-HCl (pH 8.0), 0.1 mM EDTA. Because the expression level of *PCSK2* was low, we then pre-amplified the cDNA with the TaqMan PreAmp Master Mix kit (Life Technologies, Zug, Switzerland) with 14 cycles and then performed quantitative PCR using an ABI Prism 7500 and ABI 7900 HT Sequence Detection System (Life Technologies, Zug, Switzerland). Ribosomal protein L 13 (*RPL13*), hypoxanthine phosphoribosyl-transferase-1 (*HPRT1*) and eukaryotic translation elongation factor 1A (*EEF1A*) genes were used as reference to normalize gene levels (Table S1). TaqMan probes and primers were designed and synthesized by Microsynth (Balgach, Switzerland) and Eurofins MWG Operon (Ebersberg, Germany). BLASTN searches and PCR fragment sequencing were used to check the gene specificity. Seventy-five individuals were tested in duplicate (ABI 7500) or triplicate (ABI 7900HT) in qPCR using 1x qPCR Mastermix plus low Rox (Eurogentec SA, Sereing, Belgium), in 20 / 10 ul with 2 /1 ul of pre-amplified cDNA diluted 10x depending on the qPCR machine, ABI 7500 and ABI 7900HT, respectively. For all the genes, 900 nM of each primer and 300 nM of each probe were used. When Ct values for duplicates or triplicate differed by more than 15 %, the qPCR was repeated. PCR efficiencies were between 95 and 105 %: *PCSK2*: 104.0 %, *EEF1A*: 100.1 %, *HPRT1*: 95.3 %, *RPL13*: 100.9 %. Aliquots of 3 qPCR samples were used to control for inter-plates variation. QbasePlus 2.4 software (Biogazelle, Zwijnaarde, Belgium) was used to calculate expression of *PCSK2* relative to the reference genes. M values (± CV) of the G-norm factors for *HPRT1*, *EEF1A*, *RPL13* were 0.375 ± 0.156, 0.392 ± 0.178, 0.303 ± 0.091, respectively.

**Supplementary References**

1. Medina L, Reiner A: **Do birds possess homologues of mammalian primary visual, somatosensory and motor cortices?** *Trends Neurosci*  2000, **23:**1-12.
2. Tobler I, Borbély AA: **Sleep and EEG spectra in the pigeon (*Columba livia*) under baseline conditions and after sleep-deprivation.** *J Comp Physiol- A* 1988, **163:**729-738.
3. Dewasmes G, Cohen-Adad F, Koubi H, Le Maho Y: **Polygraphic and behavioral study of sleep in geese: existence of nuchal atonia during paradoxical sleep.** *Physiol Behav* 1985, **35:**67-73.
4. Lesku JA, Meyer LC, Fuller A, Maloney SK, Dell'Omo G, Vyssotski AL, Rattenborg NC: **Ostriches sleep like platypuses.** *PLoS One* 2011, **6:**e23203.
5. Martinez-Gonzalez D, Lesku JA, Rattenborg NC: **Increased EEG spectral power density during sleep following short-term sleep deprivation in pigeons (*Columba livia*): evidence for avian sleep homeostasis.** *J Sleep Res* **17:**140-153.
6. Lesku JA, Vyssotski AL, Martinez-Gonzalez D, Wilzeck C, Rattenborg NC: **Local sleep homeostasis in the avian brain: convergence of sleep function in mammals and birds?** *Proc Biol Sci* 2011, **278:**2419-2428.

**Supplementary Table**

**Table S1** Quantitative PCR primers and probes used to measure *PCSK2* expression in feather follicles.

**Supplementary Figure**

**Figure S1** Representative EEG and accelerometry recordings showing wakefulness, non-REM sleep, and REM sleep. Eight consecutive minutes are shown with 1 minute per panel (a-h). Supplementary Video S1 shows the corresponding behavior. The first minute starts with the owlet preening (a), as reflected in the high-frequency oscillations in the accelerometer recordings and corresponding EEG artifacts. The bird then spends an extended period (>3 min) looking around the box (a-e). The small abrupt changes in the accelerometer recordings correspond to head movements. Low-amplitude, high-frequency EEG activity indicative of wakefulness is evident between the head movements. Upon falling asleep (e), the rapid head movements stop, the eyes close, and the EEG shows high-amplitude, low-frequency waves indicative of non-REM sleep. The remaining panels (f-h) show alternations between non-REM and REM sleep. During REM sleep the EEG shows wakefulness-like activity, but the head remains still or falls gradually, and the birds eyes remain closed. Similar behavior occurs in owlets without the EEG logger on their head (see bird on the right in Video S1 at 16:27:52).

**Figure S2** EEG power density for wakefulness, non-REM sleep, and REM sleep. Power is plotted at the start of each 0.4 Hz bin and expressed as a percentage of the 24-h non-REM sleep average across all frequency bins for the (a) left and (b) right hemispheres (*n* = 48 and 18, respectively). The three black lines mark significant differences (*p* < 0.05; two-tailed *t*-tests) between wakefulness and non-REM sleep (W vs N), wakefulness and REM sleep (W vs R), and non-REM and REM sleep (N vs R). Note that the Y-axis is on a log^10^ scale; i.e., peak non-REM sleep power (2 Hz bin) was over 4.5 times greater than that for wakefulness.

**Figure S3** Mean time spent in each state for all owls recorded on a given julian day and the corresponding mean temperature.

**Video S1** Eight minute video corresponding to the EEG and accelerometry recordings shown in Figure S1. The bird is positioned in the left half of the frame. A second bird can be seen in the right half of the frame.

**Figure S1**

**Figure S1 (continued)**

**Figure S1 (continued)**

**Figure S1 (continued)**

**
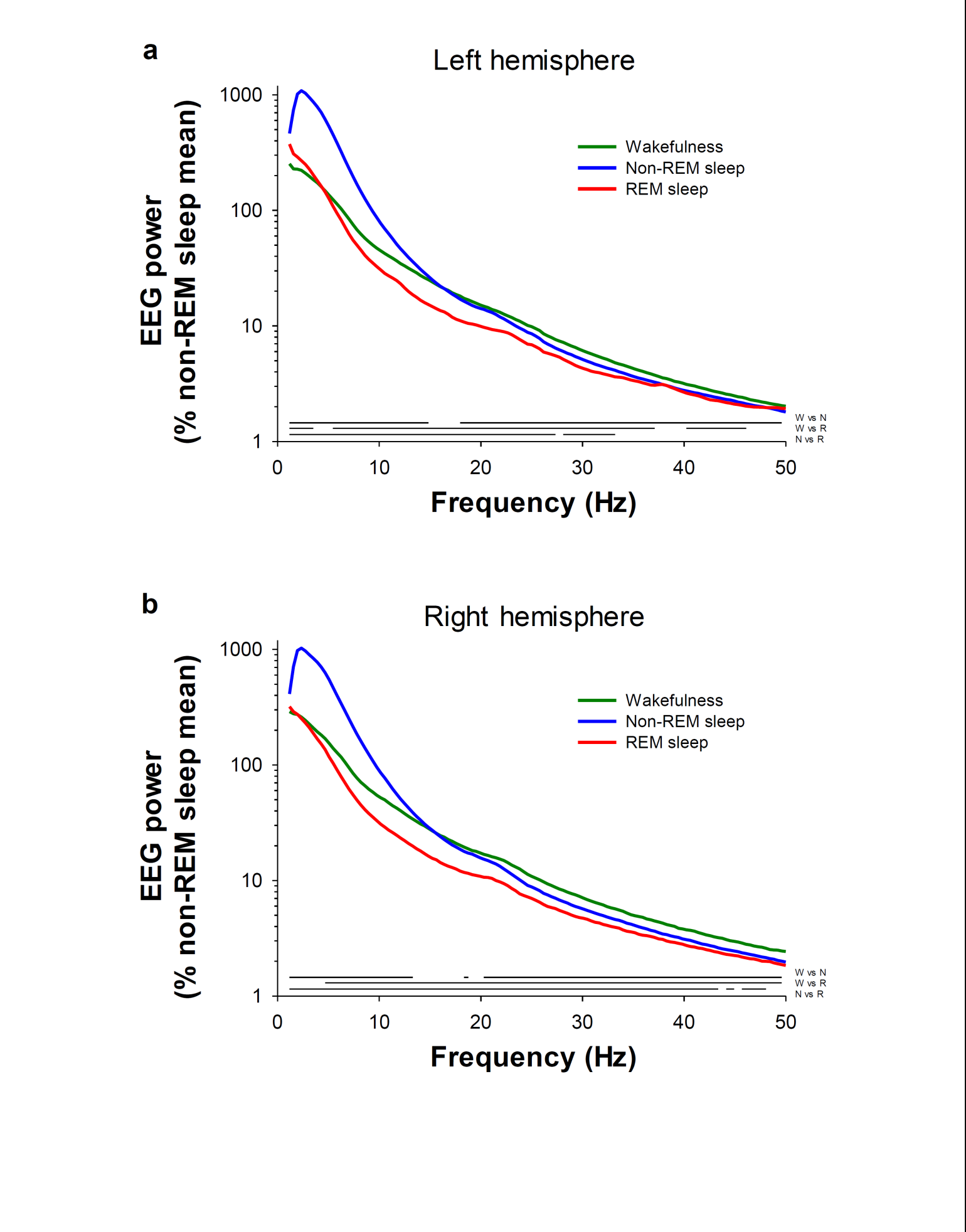
**

**Figure S2**

**Figure S3**
